# Correction: Psychedelic therapeutics in psychiatric conditions

**DOI:** 10.1038/s41386-026-02411-4

**Published:** 2026-04-16

**Authors:** Philip D. Harvey, Charles B. Nemeroff

**Affiliations:** 1 University of Miami Miller School of Medicine, Miami, FL USA; 2 Bruce W. Carter Medical Center, Miami VA Healthcare System, Miami, FL USA; 3 Dell Medical School, University of Texas at Austin, Austin, TX USA

**Keywords:** Drug delivery, Drug discovery

Correction to: *Neuropsychopharmacology* 10.1038/s41386-026-02335-z, published online 16 January 2026

“Adapted from Mian et al. (2025), with permission.” needs to be added to Fig. 1 caption. Thus, the correct Fig. 1 caption reads: Fig. 1 Thereapeutic Intent, legality and structure. Adapted from Mian et al. (2025), with permission. For instance, a medication that is approved by the FDA for prescription by physicians for a specific condition, manufactured under supervision, and dispensed by a registered pharmacy would have the highest levels of all three constructs.

The original article has been corrected.

